# *Pseudomonas aeruginosa*-derived extracellular vesicles enhance macrophage aerobic glycolysis that fuels inflammation

**DOI:** 10.3389/fmicb.2025.1619101

**Published:** 2025-07-17

**Authors:** Chun Li, Jing Peng, Lihua Xiao, Haiying Wu, Jing Chen, Nian Chen

**Affiliations:** ^1^Department of Clinical Laboratory, The Second Affiliated Hospital, Hengyang Medical College, University of South China, Hengyang, China; ^2^Department of Medical Aesthetic, The Fourth People's Hospital of Yongzhou City, Yongzhou, China; ^3^Center of Burn & Plastic and Wound Healing Surgery, The First Affiliated Hospital, Hengyang Medical College, University of South China, Hengyang, China

**Keywords:** *Pseudomonas aeruginosa*, aerobic glycolysis, toll-like receptor, outer membrane vesicle, inflammation

## Abstract

Outer membrane vesicles (OMVs) derived from *Pseudomonas aeruginosa* drive inflammation by metabolically reprogramming macrophages to favor aerobic glycolysis. This study shows that OMVs trigger this metabolic shift via Toll-like receptors 2 and 4 (TLR2/4)-dependent activation of the phosphoinositide 3-kinase (PI3K)/protein kinase B (Akt) signaling pathway. OMV-stimulated macrophages exhibited increased glucose uptake, lactate production, and expression of key glycolytic enzymes, resulting in a higher extracellular acidification rate and a lower oxygen consumption rate. Inhibition of the PI3K/Akt pathway reversed these metabolic changes. Crucially, this metabolic reprogramming was required for OMV-induced secretion of pro-inflammatory cytokines, as inhibition of glycolysis via 2-deoxy-D-glucose treatment attenuated the inflammatory response both *in vitro* and *in vivo*. These findings reveal that *P. aeruginosa* OMVs control metabolism in macrophages through the TLR2/4-PI3K/Akt axis to promote a pro-inflammatory state and identifies glycolysis as a potential therapeutic target for bacteria-associated inflammatory diseases.

## 1 Introduction

*Pseudomonas aeruginosa* is the most common opportunistic nosocomial pathogen. While the immune systems of most individuals effectively ward off infection, immunocompromised individuals may experience severe complications including septic shock, sepsis, tissue necrosis, and death (Salmanov et al., 2024; Hajdu et al., 2024). Inadequate sterilization and unsanitary practices during medical aesthetic procedures, such as tattooing and piercing, can lead to *P. aeruginosa* infection, which manifests as fever, pain, swelling, localized erythema, and pus discharge at the surgical site (Gilson et al., 2015; MacPherson et al., 2017). Exploring the pro-inflammatory mechanisms of *P. aeruginosa* could contribute to the development of better prevention and treatment strategies against these infections.

Studies have shown that *P. aeruginosa* triggers host inflammation via multiple molecular mechanisms (Ayilam Ramachandran et al., 2024). *P. aeruginosa* produces exotoxins and intracellular proteases that can induce host cells to release cytokines such as IL-1β and TNF-α, which can facilitate the development of inflammation (Jouault et al., 2022; Harder et al., 2000). In addition, the surface lipopolysaccharides (LPS) of *P. aeruginosa* can activate inflammation via toll-like receptors (TLR) (Litman et al., 2024; Zhou et al., 2014). Recently, *P. aeruginosa* has been shown to create outer membrane vesicles (OMVs), which are tiny round objects that originate from the bacterial outer membrane (Bi et al., 2024). These OMVs include diverse virulence factors, such as LPS, proteases, and short RNAs, which can interact with host cells, contribute to the development of infections, and increase the polymyxin B resistance of *P. aeruginosa* (Bitto et al., 2017; Chen et al., 2024; Xie et al., 2024; Koeppen et al., 2016). OMVs from *Porphyromonas gingivali*s have been reported to activate inflammasomes and induce pyroptotic cell death in macrophages (Fleetwood et al., 2017). However, previous studies on pro-inflammation have primarily concentrated on traditional immune signaling pathways in leukocytes, overlooking the influence of the metabolic state of host cells and leukocytes on the inflammatory response. Therefore, investigating the interactions between OMVs and the host immune system from a metabolic perspective could provide deeper insights into the pro-inflammatory mechanisms of OMVs.

During the early stages of pathogenic infection, macrophages undergo a glycolytic switch, which is characterized by increased glucose uptake and glycolysis (Jing et al., 2020; Kelly and O'Neill, 2015). This metabolic shift is necessary for the rapid proliferation and development of immune cells, providing energy and metabolic intermediates necessary for nucleotide and protein synthesis and other biosynthetic pathways required for immune cell function (Xiao et al., 2024; Yang et al., 2024). However, whether *P. aeruginosa*-secreted OMVs can induce macrophage metabolic switch and the underline mechanism remains underexplored.

In this research, we investigated the effects of *P. aeruginosa*-derived OMVs on macrophages *in vitro*. Our findings revealed that OMVs promote aerobic glycolysis in murine macrophages through a mechanism that is dependent on TLR2- and TLR4-mediated phosphoinositide 3-kinase (PI3K)/protein kinase B (Akt) activation. Inhibition of aerobic glycolysis resulted in a significant decrease in OMV-induced cytokine levels, similar to the severity of *in vivo* inflammation. These findings indicate that OMVs serve a vital function as pro-inflammatory factors in *P. aeruginosa* infection.

## 2 Materials and methods

### 2.1 Chemicals and antibodies

Glycolysis Stress Test Kit and Cell Mito Stress Test Kit were provided by Agilent (CA, USA). 2-deoxy-D-glucose (2-DG) and LY294002 were purchased from Sigma-Aldrich (MO, USA). Anti-phosphorylated Akt and total Akt monoclonal antibodies were obtained from Cell Signaling Technology (MA, USA). TNF-α, IL-1β, and IL-6 cytokine assay kits were provided by Wuhan Huamei Biotechnology (Wuhan, China).

### 2.2 *P. aeruginosa* culture and isolation of OMVs

OMVs were isolated and characterized as described previously (Li et al., 2024). Briefly, *P. aeruginosa* strain PAO1 (ATCC 10145) was cultured in Luria-Bertani medium with agitation until an optical density of 1.2 was reached. Subsequently, bacterial cultures were subjected to centrifugation at 10,000 × *g* for 20 min to separate bacterial cells, followed by filtration of the resulting supernatant through a 0.22 μm filter. The filtered supernatant was ultracentrifugated at 200,000 × *g* for 2 h at 4°C to pellet the OMVs. OMV pellets were resuspended in a 60% OptiPrep Density Gradient Medium (Sigma-Aldrich) and layered with 40%, 35%, 30%, or 20% OptiPrep diluted in OMV buffer. OMVs in OptiPrep layers were centrifuged at 100,000 × *g* for 16 h at 4°C. Fractions (500 μL) were taken from the top of the gradient, with OMVs residing in fractions 2–3, corresponding to 25% OptiPrep. This isolation protocol was previously validated by physical characterization methods such as transmission electron microscopy to yield purified OMVs (Li et al., 2024; Ellis et al., 2010). The obtained OMVs were quantified using a Pierce™ Bicinchoninic Acid (BCA) Protein Assay Kit (Thermo Fisher Scientific, MA, USA), according to the manufacturer's instructions. The OMV stocks were stored at −80°C, and all dosages used in the experiments are reported as the final protein concentration (μg/mL).

### 2.3 Macrophage culture and OMV stimulation

TLR4^−/−^ (C57BL/6Smoc-Tlr4^em1Smoc^), TLR2^−/−^ (C57BL/6Smoc-Tlr2^em1Smoc^), TRIF^−/−^ (C57BL/6Smoc-Ticam1^em2Smoc^), and TRAM^−/−^ (C57BL/6Smoc-Ticam2^em1Smoc^) mice were purchased from Shanghai Model Organisms Center, Inc. (China). C57BL/6 mice were anesthetized with CO_2_, and the tibiae and femora were isolated to harvest bone marrow-derived macrophages (BMDMs). BMDMs were cultured in conditioned medium supplemented with 10% L929 cell culture supernatant for 6 d to induce differentiation into mature macrophages. Subsequently, the cells were counted and transferred to new plates for further experiments. For the stimulation experiments, 10^6^ BMDMs were stimulated with varying concentrations of OMVs over different time intervals.

### 2.4 Quantitative PCR

Cytokine mRNAs expression was measured via fluorescence-based quantitative PCR. Initially, total RNA was isolated using the TRIzol reagent (Invitrogen, MA, USA) and reverse transcribed to cDNA using a commercial kit (TaKaRa, Japan). The cDNA was then used as a template for quantitative PCR using a fluorescence quantitation kit (SuperReal PreMix Plus; TIANGEN, Beijing, China) according to the manufacturer's instructions. Each reaction was performed thrice. Relative mRNA expression was determined using the comparative threshold cycle (CT) approach, in which mRNA expression was normalized to that of GAPDH.

### 2.5 Immunoblot analysis

After cellular treatment, the cells were lysed with 100 μL of a specialized lysis buffer and protease inhibitors (Roche, Switzerland). The lysates were then ultrasonicated for 10 s. The resulting supernatant was isolated after centrifugation at 20,000 × *g* for 10 min at 4°C. Following protein concentration quantification, proteins in the range of 15–35 μg were subjected to sodium dodecyl-sulfate polyacrylamide gel electrophoresis. After electro-transferring the proteins, the membrane was blocked with non-fat milk and incubated in primary antibodies overnight at 4°C. A washing step was performed, followed by the addition of a horseradish peroxidase-conjugated secondary antibody, and incubation was maintained at ambient temperature for 30 min. Visualization was achieved using a chemiluminescence detection method, after which an image was acquired.

### 2.6 Detection of glycolysis and oxidative phosphorylation

The extracellular acidification rate (ECAR) and oxygen consumption rate (OCR) were measured using a Seahorse XFe96 analyzer (Agilent). BMDMs were seeded at a density of 2.5 × 10^4^ cells/well in a Seahorse XF96 cell culture microplate and allowed to adhere overnight. After stimulation with OMVs for the indicated durations, the standard culture medium was replaced with assay-specific medium and the plate was incubated at 37°C in a non-CO_2_ incubator for 1 h to allow for temperature and pH equilibration. For the cell mitosis stress test, cells were incubated in XF Base Medium supplemented with 10 mM glucose, 2 mM L-glutamine, and 1 mM sodium pyruvate. The assay injection protocol consisted of sequential additions of the following compounds to achieve the indicated final concentrations: 1.5 μM oligomycin, 1.0 μM FCCP, and 0.5 μM rotenone/antimycin A. For the glycolysis stress test, cells were incubated in XF Base Medium supplemented with 2 mM L-glutamine. The assay injection protocol involved sequential additions of 10 mM glucose, 1.5 μM oligomycin, and 50 mM 2-DG. The instrument was programmed to perform a standard cycle of 3 min of mixing followed by 3 min of measurement for each data point. After completing the assay, the cells were lysed and the total protein content in each well was quantified using a BCA protein assay kit. The OCR and ECAR values were then normalized to the protein content (μg) for each well to account for variations in cell number.

### 2.7 Measurement of glucose consumption and lactate production

After OMV stimulation, the culture supernatant was collected and centrifugated for 5 min at 37°C. Then, the glucose and lactate concentrations in the supernatant were measured using a biochemical analyzer (Cobas 8000; Roche).

### 2.8 Determination of cytokines

Quantitative evaluation of cytokines in the culture supernatant was meticulously conducted using enzyme-linked immunosorbent assay (ELISA). The ELISA plate was pre-coated with specific antibodies against TNF-α, IL-6, and IL-1β, following the protocol of the eBioscience kit. The supernatant (100 μL) was dispensed into the respective wells, followed by incubation at 37°C for 90 min to optimize cytokine capture. After washing, biotin-conjugated antibodies complemented with horseradish peroxidase-tagged anti-biotin antibodies were added to the wells. The subsequent chromogenic reaction was facilitated using the 3,3′,5,5′-tetramethylbenzidine substrate, and absorbance was measured at a wavelength of 450 nm. Analytical quantification was performed using an established standard curve.

### 2.9 *In vivo* infection

Female C57BL/6 mice aged 6–8 weeks were obtained from SLAC (Shanghai, China). All animal-related procedures were approved by the Medical Ethics Committee of the University of South China. The mice were housed in a specific pathogen-free room and provided *ad libitum* access to food and water. For *in vivo* experiments, he mice were anesthetized with isoflurane inhalation, and 50 μg of OMVs was administered intranasally. Lung tissues were harvested 4 d after administration for subsequent analysis.

### 2.10 Statistical analysis

Data are presented as the mean ± SEM and were analyzed using one-way analysis of variance (ANOVA), followed by Tukey's *post hoc* test. Statistical significance was set at *P* < 0.05.

## 3 Results

### 3.1 OMVs induce metabolic reprogramming in macrophages

**Figure 1:**
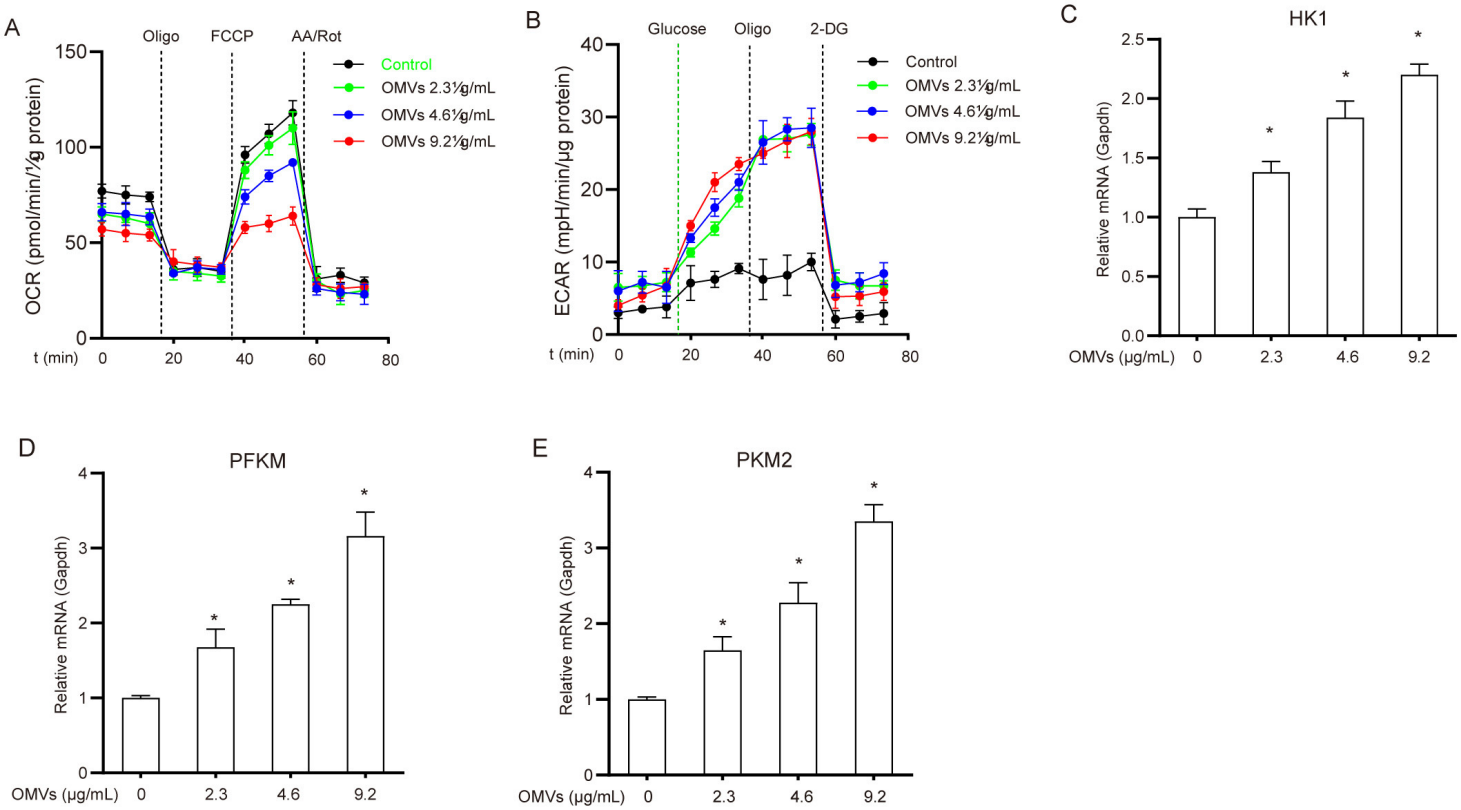
OMVs induce metabolic reprogramming in macrophages. **(A, B)** BMDMs were exposed to 9.2 μg/mL OMVs for 24 h before the addition of the indicated compounds, and OCR **(A)** and ECAR **(B)** were measured. **(C–E)** Cells were incubated with 2.3, 4.6, or 9.2 μg/mL OMVs for 24 h, and the mRNA levels of HK1, PFKM, and PKM2 were assessed via real-time PCR. Data are presented as the mean ± standard error of the mean (SEM) from three independent experiments. Statistical significance was determined using one-way ANOVA with Tukey's *post hoc* test. *P < 0.05 vs. control group (0 μg/mL).

A major early event in the immune response following pathogenic microbial infection or recognition of pathogen-associated molecular patterns (PAMPs) is the metabolic shift in immune cells from the tricarboxylic acid (TCA) cycle/oxidative phosphorylation to aerobic glycolysis (Tartey and Takeuchi, 2017). To assess the metabolic status of OMV-stimulated macrophages, we monitored the changes in ECAR and OCR. After 24 h of OMV treatment, ECAR significantly increased while OCR markedly decreased compared with the control (Figures 1A, B). Furthermore, OMV treatment upregulated hexokinase 1 (HK1), phosphofructokinase M (PFKM), and pyruvate kinase M2 (PKM2) mRNAs in a dose-dependent manner (Figures 1C–E). These results suggest that OMVs induce a metabolic shift in macrophages from the TCA cycle to aerobic glycolysis.

### 3.2 OMVs increase glucose uptake

**Figure 2:**
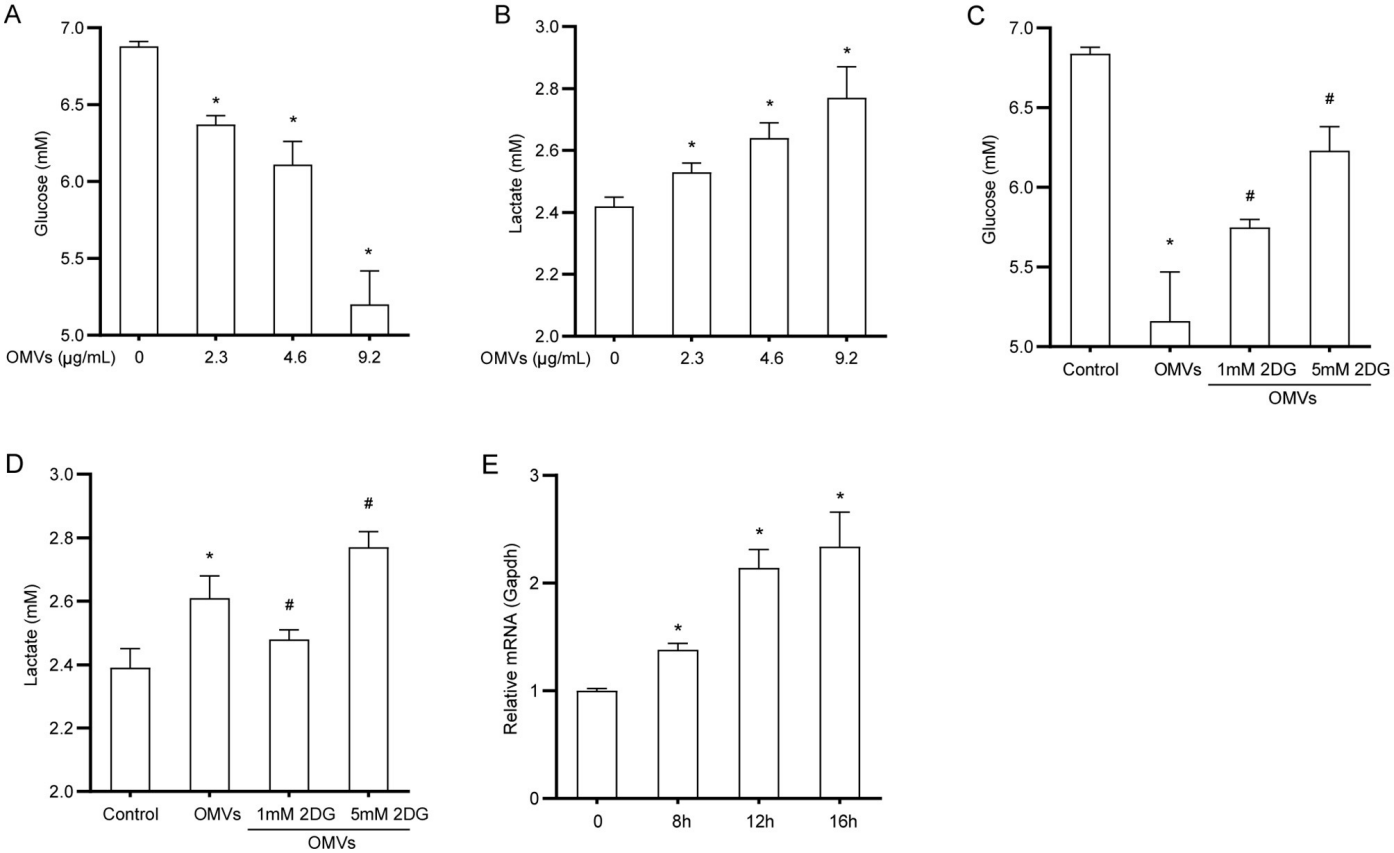
OMVs increase glucose uptake and lactate production. **(A, B)** BMDMs were stimulated with OMVs (0, 2.3, 4.6, or 9.2 μg/mL) for 24 h, and glucose and lactate concentrations in the supernatant were measured. **(C, D)** Cells were pre-treated with 2-DG for 3 h and then stimulated with 9.2 μg/mL OMVs for 24 h. Glucose and lactate concentrations were measured. **(E)** BMDMs were stimulated with 9.2 μg/mL OMVs for 0, 8, 12, or 16h, and GLUT1 mRNA was measured via real-time PCR. Data are presented as the mean ± SEM from three independent experiments. Statistical significance was determined using one-way ANOVA with Tukey's *post hoc* test. *P < 0.05 vs. control group. ^#^*P* < 0.05 vs. OMVs group.

To explore the effect of OMVs on glucose metabolism in macrophages, we first examined the metabolic dynamics of glucose and lactate in OMV-treated cells. OMV treatment led to a significant increase in glucose consumption from the medium; specifically, the highest dose of OMVs resulted in a 24% reduction in supernatant glucose levels compared with the control (Figure 2A). Consistent with enhanced glycolytic flux, this increased glucose uptake was accompanied by a corresponding increase in lactate production. The lactate concentration in the supernatant of OMV-treated macrophages increased by approximately 13% compared with that in the control group (Figure 2B). We used the glycolysis inhibitor 2-DG to confirm that these metabolic changes were due to glycolysis. To ensure that 2-DG did not cause nonspecific effects at the working concentration, we evaluated its cytotoxicity. The cell viability assay demonstrated that 2-DG did not significantly affect macrophage viability (Supplementary Figure S1). Having confirmed its safety, we found that pretreatment with 2-DG markedly reversed OMV-induced increase in glucose consumption and lactate production (Figures 2C, D). In line with these observations, the mRNA expression of the key glucose transporter 1 (GLUT1) substantially increased by approximately 2.3-fold 16 h after OMV treatment (Figure 2E). These results suggest that OMVs stimulate glucose uptake and glycolysis in macrophages, a process that can be specifically blocked by 2-DG without inducing cell toxicity.

### 3.3 OMVs induce aerobic glycolysis in macrophages via TLR2 and TLR4

**Figure 3:**
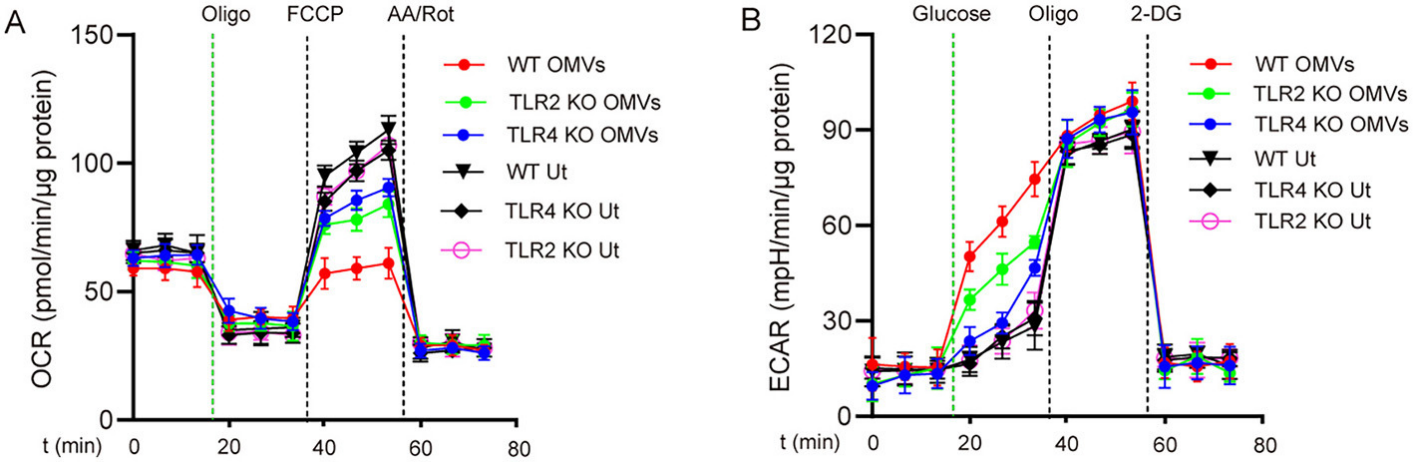
TLR2 and TLR4 are involved in OMV-induced aerobic glycolysis in macrophages. TLR2- or TLR4-knockout or wild-type BMDMs were stimulated with or without OMVs (9.2 μg/mL) for 24 h. OCR **(A)** was measured before and after sequential addition of oligomycin, FCCP, and antimycin A and rotenone (AA/Rot) or ECAR **(B)** was detected, followed by subsequent treatment with 10 mmol/L glucose, 1 μmol/L oligomycin, and 100 mmol/L 2-DG. Data are presented as the mean ± SEM from three independent experiments.

Because OMVs contain PAMPs such as LPS and lipoproteins (Zariri et al., 2016), we hypothesized that these pro-inflammatory substances promote metabolic reprogramming in macrophages through TLRs. We analyzed the effects of OMV stimulation on metabolic reprogramming in TLR2- and TLR4-deficient macrophages. Following OMV stimulation, wild-type macrophages exhibited a considerable decrease in ECAR and a significant increase in OCR compared with TLR2- or TLR4-deficient macrophages (Figures 3A, B). These results indicate that OMVs primarily mediate metabolic reprogramming in macrophages through TLR2 and TLR4.

### 3.4 TRIF and TRAM are crucial for OMV-induced aerobic glycolysis

**Figure 4:**
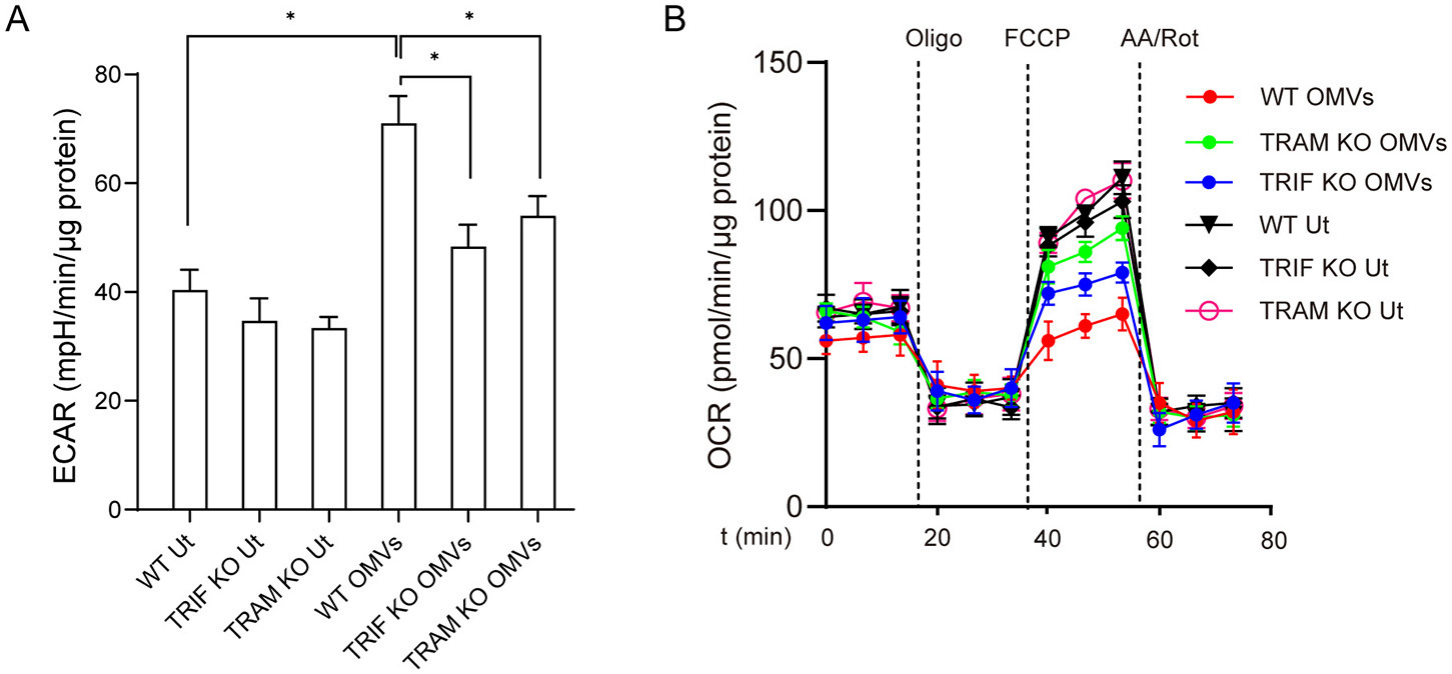
OMV-induced aerobic glycolysis is mediated by TRIF and TRAM. Wild-type or TRIF- or TRAM-knockout BMDMs were incubated with OMVs (9.2 μg/mL) for 24 h, and ECAR **(A)** and OCR **(B)** were measured. Data are presented as the mean ± SEM from three independent experiments. Statistical significance was determined using one-way ANOVA with Tukey's *post-hoc* test. *P < 0.05, as indicated.

TRIF and TRAM are essential downstream adaptors of TLR2 and TLR4, which are pivotal for mediating immune responses (Stack et al., 2014). To investigate the role of TRIF and TRAM in OMV-mediated aerobic glycolysis, we used bone marrow-derived macrophages from TRIF and TRAM knockout mice. The results showed that TRIF- or TRAM-deficient macrophages had a significantly reduced metabolic remodeling capacity compared with wild-type macrophages after OMV stimulation (Figures 4A, B), highlighting the important roles of TRIF and TRAM in OMV-induced metabolic changes.

### 3.5 TLR2 and TLR4 mediate aerobic glycolysis via the PI3K/Akt pathway

**Figure 5:**
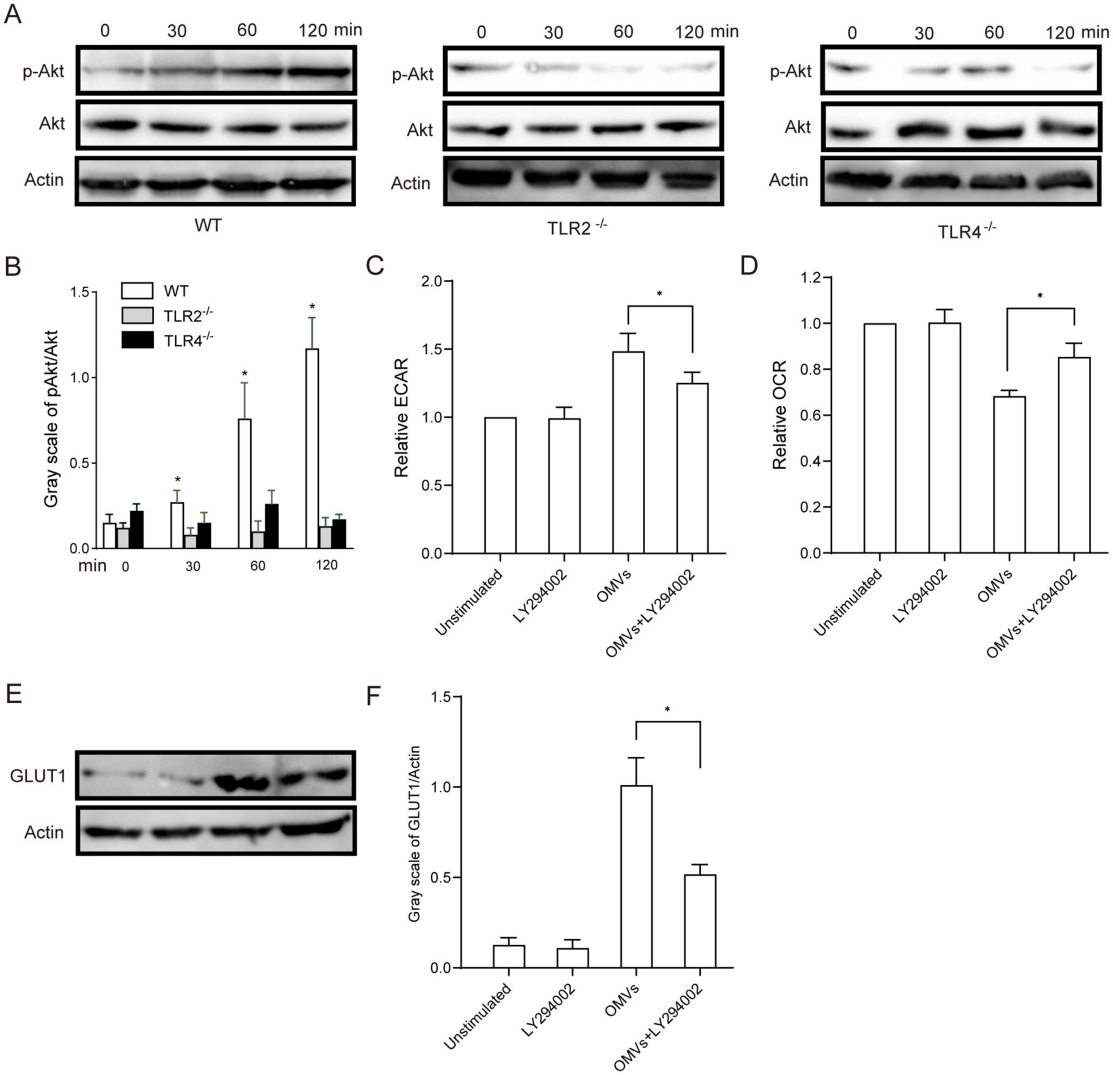
OMV-activated aerobic glycolysis is regulated by TLR2- and TLR4-mediated PI3K/Akt activation. **(A, B)** TLR2- or TLR4-knockout BMDMs were stimulated with 9.2 μg/mL OMVs for 0, 30, 60 or 120 min, and phosphorylated Akt was detected via western blotting. **(C, D)** BMDMs were preincubated with 20 μM of the PI3K/Akt inhibitor LY294002 for 30 min, and the ECAR and OCR were measured via extracellular flux analysis. **(E, F)** BMDMs were pre-treated with LY294002 (20 μM) for 30 min and then stimulated with OMVs (9.2 μg/mL) for 12 h. GLUT1 protein expression was detected via western blotting. Data are presented as the mean ± SEM from three independent experiments. Statistical significance was determined using one-way ANOVA with Tukey's *post hoc* test. *P < 0.05 vs. indicated groups or 0 min.

Western blotting analysis revealed that OMV stimulation induced Akt phosphorylation in macrophages after 30 min, which persisted for more than 2 h. In contrast, OMV-induced Akt phosphorylation was significantly delayed in the TLR2- and TLR4-deficient macrophages (Figures 5A, B). To confirm the functional role of the relevant pathway, we pre-treated macrophages with the PI3K/Akt inhibitor LY294002. As shown in Figures 5C, D, LY294002 pre-treatment significantly attenuated the metabolic effects of OMVs, mitigating the increase in ECAR and decreasing OCR. Given that the PI3K/Akt pathway is a key regulator of glucose uptake, often by controlling the expression and translocation of glucose transporters, such as GLUT1, we sought to establish this mechanistic link. Western blotting was performed to assess GLUT1 protein levels following Akt inhibition. OMV stimulation robustly increased the expression of GLUT1, an effect that was almost completely abrogated by pre-treatment with LY294002 (Figures 5E, F). Taken together, these results strongly suggest that OMVs activate TLR2 and TLR4, leading to the induction of aerobic glycolysis in macrophages via the PI3K/Akt pathway, which includes critical upregulation of the glucose transporter GLUT1.

### 3.6 OMVs exert pro-inflammatory effects via aerobic glycolysis

**Figure 6:**
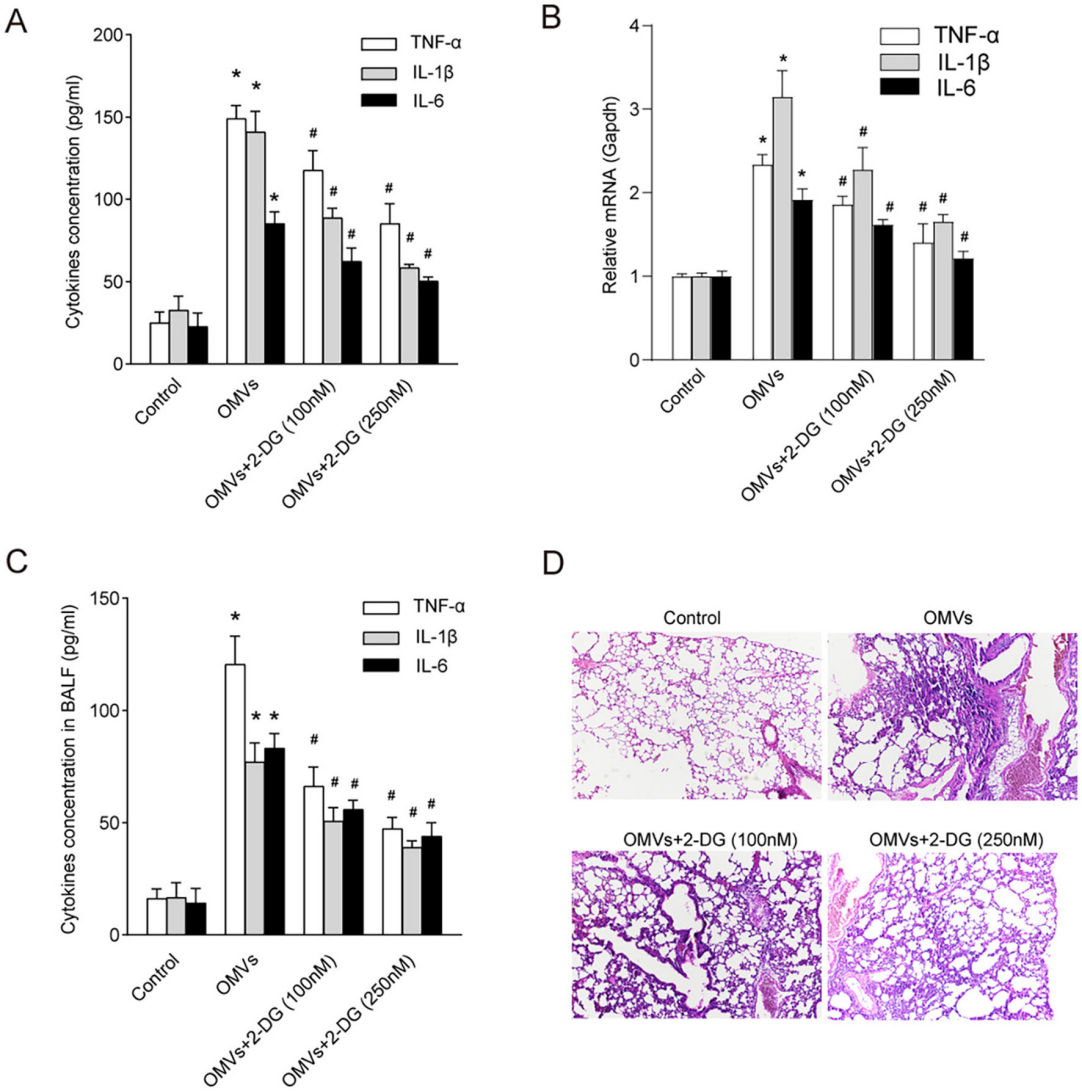
Inhibition of aerobic glycolysis abrogates OMV-induced inflammatory responses *in vitro* and *in vivo*. **(A)** BMDMs were pre-treated with 100 or 250 nM 2-DG for 3 h and then stimulated with OMVs (9.2 μg/mL). After 24 h, the concentrations of pro-inflammatory cytokines in the culture supernatant were measured using ELISA. **(B–D)** C57BL/6 mice (*n* = 6–8 per group) were nasally administered OMVs (50 μg) with or without 250 nM 2-DG. After 24 h of stimulation, the mice were euthanized. **(B)** The relative mRNA expression levels of Tnf-α, Il-1β, and Il-6 in lung tissue homogenates were measured via real-time PCR. **(C)** The concentrations of TNF-α, IL-1β, and IL-6 in the BALF were measured via ELISA. **(D)** Lung tissues were subjected to histological analysis by H&E staining. All experiments were performed in triplicate, and data are presented as the mean ± SEM. Statistical significance was determined using one-way ANOVA with Tukey's *post hoc* test. *P < 0.05 vs. control group; ^#^*P* < 0.05 vs. OMV-only group.

Enzymes and metabolites of aerobic glycolysis play crucial roles in the regulation of inflammation (Soto-Heredero et al., 2020). To investigate whether OMV-induced aerobic glycolysis is responsible for the secretion of pro-inflammatory cytokines, we pre-treated macrophages with the glycolysis inhibitor 2-DG. Expectedly, our results showed that OMVs significantly increased the secretion of TNF-α, IL-1β, and IL-6 in macrophages, which was markedly attenuated by the inhibition of glycolysis (Figure 6A). To validate these findings *in vivo* and assess their translational relevance, we intranasally administered OMVs to C57BL/6 mice with or without 2-DG. We first quantified the expression of key inflammatory cytokine genes in the lung tissue. OMV administration led to a significant upregulation of *Tnf-*α, *Il-1*β, and *Il-6* mRNA in the lungs, which was markedly suppressed by co-treatment with 2-DG (Figure 6B). Correspondingly, the protein concentrations of TNF-α, IL-1β, and IL-6 in the BALF were substantially elevated in OMV-challenged mice, whereas co-administration with 2-DG significantly reduced these cytokine levels (Figure 6C). Our *in vivo* experiments validated these results. Histological analysis of the lung tissue showed that mice in the control group exhibited normal, clear alveolar structures. In contrast, intranasal administration of OMVs led to severe pulmonary inflammation, characterized by massive infiltration of inflammatory cells (such as neutrophils and macrophages) into the alveolar spaces, significant thickening of the alveolar septa, and evidence of tissue damage. This OMV-induced lung pathology was markedly ameliorated in mice co-treated with 2-DG. The 2-DG-treated groups showed significantly reduced cellular infiltration and better preserved lung architecture in a dose-dependent manner, confirming that inhibition of glycolysis can mitigate OMV-induced inflammation *in vivo* (Figure 6D). Collectively, these *in vitro* and *in vivo* results indicate that OMVs promote pro-inflammatory cytokine secretion by inducing aerobic glycolysis, which is a critical driver of subsequent inflammatory pathology.

## 4 Discussion

OMVs are integral to the pathogenesis of *P. aeruginosa*. A critical aspect of *P. aeruginosa* OMVs in pathogenesis is their ability to influence the host immune response (Koeppen et al., 2016). *P. aeruginosa* also utilizes OMVs to subvert the host immune system and induce chronic infections. For instance, the OMV-associated virulence factor Cif promotes degradation of the cystic fibrosis transmembrane conductance regulator in airway epithelial cells, impairing mucociliary clearance and increasing bacterial colonization (MacEachran et al., 2007). OMVs are involved in the transfer of small regulatory RNAs to host cells. These sRNAs modulate host cell functions such as attenuating cytokine secretion, thus dampening the immune response and facilitating persistent infection (Magaña et al., 2023). Understanding these mechanisms highlights the complex role of OMVs in the pathogenicity of *P. aeruginosa* and offers potential avenues for developing targeted therapies to combat infections caused by this opportunistic pathogen. In this study, we report that OMVs are key pro-inflammatory agents that can induce metabolic reprogramming in macrophages *in vitro*. This metabolic shift is dependent on TLR2 and TLR4 activation and ultimately affects cytokine secretion and inflammatory responses. It not only fuels immune cells but also drives the inflammatory response, highlighting the intricate interplay between metabolism and immune regulation during bacterial infection.

The early immune response to microbial infection involves a metabolic transition from oxidative phosphorylation to aerobic glycolysis in the immune cells (Guo et al., 2021; O'Neill and Pearce, 2016). Our findings support this paradigm by showing significantly increased ECAR and decreased OCR in OMV-stimulated macrophages as well as increased lactate and decreased glucose concentrations in the culture supernatant after treatment with OMVs, indicating enhanced glycolysis and reduced mitochondrial respiration, respectively. Considering that increased glucose metabolism is often accompanied by changes in glucose transporter expression, we measured the expression of GLUT1, the major transporter of glucose metabolism. Our results confirmed that GLUT1 expression increased after OMV stimulation, thereby continuously providing energy for glucose metabolism in host cells. This aligns with the results of previous studies highlighting the importance of aerobic glycolysis in immune cell activation and effector functions (Pearce and Pearce, 2013; Palsson-McDermott and O'Neill, 2020). Czyz et al. reported that OMVs from *Brucella* species promoted glycolysis in host cells, leading to increased lactate production (Czyz et al., 2017). Similarly, OMVs from other pathogenic microorganisms have been shown to regulate host cell metabolism via TLR signaling pathways, thereby influencing immune responses and inflammatory processes (Lee et al., 2009; Jun et al., 2013). These findings indicate that glycolysis is a common mechanism by which OMVs stimulate macrophages to exert pro-inflammatory effects.

TLRs, particularly TLR2 and TLR4, recognize OMVs in certain pathogens (Kawai and Akira, 2010). Consistent with this, we observed that TLR2- and TLR4-deficient macrophages exhibited attenuated aerobic glycolysis in response to OMVs, highlighting the central role of these receptors in the sensing and transduction of OMV-derived signals. This is consistent with studies showing the involvement of TLR signaling in metabolic regulation and immune cell function (Sasaki and Firtel, 2006). Upon ligand binding and activation, TLRs trigger dual signaling pathways: one involving Mal and MyD88, leading to NF-κB activation and the transcription of pro-inflammatory cytokines, and the other allowing TLR2 and TLR4 to enter the cell and form endosomes, where TRAM recruits TRIF, facilitating interferon expression (Nilsen et al., 2015). Our study further demonstrates that aerobic glycolysis induced by OMV stimulation is associated with TRAM and TRIF, confirming the crucial role of these downstream adaptors of TLR2 and TLR4 in mediating pro-inflammatory signal transduction. This suggests the involvement of the interferon pathway in metabolic changes in macrophages. Additionally, our findings are supported by the evidence that OMVs transfer nucleic acids to host cells, resulting in the untimely induction of type I interferon (Liu et al., 2022; Di et al., 2023). The activation of IRF signaling by OMVs plays a role in antiviral immune responses and host defense mechanisms.

Downstream signaling events triggered by TLR activation, particularly the PI3K/Akt pathway, have been extensively studied in the context of the modulation of glucose uptake through several mechanisms, including increased vesicle transport and GLUT1 phosphorylation (Shan et al., 2024; Fontana et al., 2024). Our findings reveal the dependence of OMV-induced Akt phosphorylation on TLR2 and TLR4, further underscoring the intricate crosstalk between immune signaling pathways and cellular metabolism (Palsson-McDermott and O'Neill, 2020). The inhibition of the PI3K/Akt pathway reverses the metabolic effects of OMVs, highlighting the therapeutic potential of targeting this pathway to modulate inflammatory responses. Expectedly, our study demonstrated that OMV-induced aerobic glycolysis was linked to pro-inflammatory cytokine expression. Importantly, inhibition of glycolysis attenuated cytokine release *in vivo* and *in vitro*, highlighting the therapeutic potential of targeting aerobic glycolysis to mitigate OMV-induced inflammation.

In conclusion, our data provide new insights into the immunomodulatory effects of OMVs and their ability to control macrophage metabolism to promote inflammation. Our findings lay the groundwork for the development of targeted therapies for bacteria-induced inflammatory disorders by elucidating the complex signaling networks involved in OMV-induced metabolic reprogramming. Further research is warranted to unravel the full spectrum of OMV-host interactions and their implications for immune regulation and disease pathogenesis.

## Data Availability

The original contributions presented in the study are included in the article/Supplementary material, further inquiries can be directed to the corresponding author.
