# Supplementary material for: Pseudomonas aeruginosa-derived extracellular vesicles enhance macrophage aerobic glycolysis that fuels inflammation
Source: Front Microbiol. 2025 Jul 17;16:1619101. doi: 10.3389/fmicb.2025.1619101 (PMC12310700; doi:10.3389/fmicb.2025.1619101)
Supplement: Supplementary file 1 [file Data_Sheet_1.docx]

**Li et al. Supplemental Materials**

**Materials**

The LDH-Glo™ Cytotoxicity Assay was purchased from Promega (Madison, WI, USA)，2-deoxy-D-glucose (2-DG) was obtained from Sigma-Aldrich (St. Louis, MO).

**Methods**

**Cell Viability Assay**

To assess the cytotoxicity 2-DG, cell viability was determined by measuring the release of lactate dehydrogenase (LDH) from BMDMs. The LDH-Glo™ Cytotoxicity Assay was used according to the manufacturer's protocol. Briefly, BMDMs were seeded in 96-well plates and treated with various concentrations of 2-DG for 24 hours. For each experiment, control wells were included to determine spontaneous LDH release (from untreated cells) and maximum LDH release (from cells treated with the provided lysis buffer). After the 24-hour incubation, LDH release was measured according to the manufacturer's protocol. The percentage of LDH leakage was calculated using the following formula: LDH Leakage (%) = [(Experimental Release-Spontaneous Release)/(Maximum Release-Spontaneous Release) ]×100.

**Statistical analysis**

All data are presented as the mean ± standard error of the mean (SEM) from at least three independent experiments. Statistical significance was determined using a one-way analysis of variance (ANOVA) followed by Tukey's post hoc test for multiple comparisons. A p-value of less than 0.05 was considered statistically significant.

**Supplementary figures**


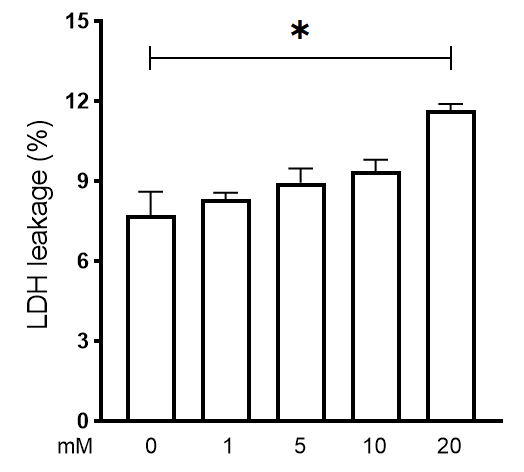


**Figure S1. Effect of 2-DG on Macrophage Viability.** BMDMs were treated with increasing concentrations of 2-DG (0, 1, 5, 10, and 20 mM) for 24 hours. Cell viability was assessed by measuring LDH release into the supernatant. Data are presented as the percentage of LDH leakage (Mean ± SEM) from three independent experiments, relative to a maximum lysis control. P < 0.05 as compared with the control group (0 mM).
